# Induction mechanism of cigarette smoke components (CSCs) on dyslipidemia and hepatic steatosis in rats

**DOI:** 10.1186/s12944-022-01725-8

**Published:** 2022-11-08

**Authors:** Jian Ge, Wei-jia Xu, Hai-feng Chen, Zong-hua Dong, Wei Liu, Fu-zhao Nian, Jun Liu

**Affiliations:** 1 College of Life Sciences, China Jiliang University, 258 XueYuan Street, XiaSha Higher Education Zone, Hangzhou, 310018 Zhejiang Province People’s Republic of China; 2 College of Tobacco Science, Yunnan Agricultural University, Kunming, 650201 Yunnan China; 3 Institute of Microbiology, Zhejiang Academy of Agricultural Sciences, Hangzhou, 310021 Zhejiang China

**Keywords:** Cigarette smoke components (CSC), Dyslipidemia, Gut microbiota, Serum exosomes, miRNA regulation

## Abstract

**Objective:**

The purpose of this study was to explore the effect of cigarette smoke component (CSC) exposure on serum lipid levels in rats and the underlying molecular mechanism.

**Methods:**

Male SPF-grade SD rats were randomly divided into a control group and a CSC exposure group, with the CSC group being exposed to CSC for 6 weeks. RT–PCR and Western blotting methods were used to detect lipid metabolism gene expression in rats, and 16S RNA gene sequencing was used to detect the gut microbiota in the rat cecum. Rat serum exosomes were prepared and identified, and the interaction of exosomal miR-291a-3p and miR-126a-5p with AMPK and CYP7A1 was detected by a dual luciferase reporter gene assay (DLRG).

**Results:**

Serum indicators, including cholesterol levels and trimethylamine oxide (TMAO) content, were significantly affected in the CSC exposure group compared with the control group (*P* < 0.05), and the expression levels of adenylate-activated protein kinase (AMPK), acetyl-coenzyme A carboxylase (ACC) and HMG-CoA reductase (HMG-CoAR) genes were significantly increased (*P* < 0.05) in the liver, while the expression level of cholesterol 7α-hydroxylase (CYP7A1) was markedly decreased (*P* < 0.01). 16S rRNA gene sequencing of the gut microbiota in the rat cecum showed that the abundance of Firmicutes in the CSC group increased significantly at the phylum level, while the abundances of Bacteroidota and Spirochaetota were reduced significantly (*P* < 0.01). The relative abundance of Romboutsia, Turicibacter, and Clostridium sensu stricto increased significantly (*P* < 0.01), and the relative abundance of Prevotella, Muribaculaceae_norank, Lachnospiraceae NK4A136 group, Roseburia, Treponema, and Ruminococcus significantly decreased (*P* < 0.01) at the genus level. In addition, the exosome miR-291a-3p and miR-126a-5p levels were markedly regulated by CSC exposure (*P* < 0.01). The interactions of miR-291a-3p and miR-126a-5p with AMPK and CYP7A1 mRNA were also validated by the DLRG method.

**Conclusions:**

In summary, the rat dyslipidemia induced by CSC exposure may be related to the interference of gut microbiota structure and interaction of miRNAs from serum exosomes with target mRNAs, which further regulated AMPK-ACC/CYP7A1 signaling in rats.

## Introduction

Today, smoking is still common in most countries in the world and has caused serious public health problems in both developed and developing countries. In addition to cancer, it can lead to a variety of diseases, including cardiovascular diseases, digestive system diseases, and central nervous system diseases [1]. Numerous surveys have found that there are currently more than 316 million smokers in China, nearly 740 million nonsmokers suffer from second-hand smoke exposure, and more than 1 million people die from smoking-related diseases every year [2]. Recently, some clinical scholars at home and abroad have pointed out that smoking can significantly cause abnormal lipid metabolism and affect the occurrence of diabetes and its chronic vascular complications [3, 4], and dyslipidemia has become an independent risk factor for fatty liver [ [5]]. Tobacco smoking induces cardiovascular mitochondrial oxidative stress, promotes endothelial dysfunction, and enhances hypertension [6]. In addition, smoking has also exerted a significant influence on alcoholic fatty liver [7]. The risk of alcoholic cirrhosis in smokers is 3.45 times that of nonsmokers [8]. However, the underlying mechanism of abnormal lipid metabolism induced by smoking has not been fully elucidated.

It is well known that smoking gas during inhalation is divided into mainstream smoke and side stream smoke [9]. The mainstream smoke gas enters the smoker’s mouth, a portion of smoke gas enters the lungs through the larynx, and another portion enters the digestive tract through the pharynx [10]. However, it is difficult to develop toxicity research on smoking in experimental animals. Because animals did not take the initiative to inhale the smoking gas, it was difficult to carry out in-depth research on experimental animals [11]. Considering that regardless of whether the mainstream smoke was inhaled through the larynx and pharynx into the respiratory tract or digestive tract, it was the water-soluble components of the cigarette smoke gas that entered the epithelium or blood circulation to cause toxicity through the mucous layer and biomembrane (respiratory tract epithelial cell membrane or digestive tract epithelial cell membrane) or affected the gut microbiota community. Then, the most liposoluble constituents of cigarette smoke gas were exhaled outside the body, which was called “second-hand smoke” [12, 13]. Therefore, cigarette smoke components (CSCs) were prepared and used to study the effect on lipid metabolism.

Currently, there are a large number of retrospective investigation reports and clinical sampling analyses (such as blood, feces and urine from smokers) about the consequences of smoking [14, 15]. It contributes significantly to the morbidity and mortality of cardiovascular diseases. As a potential mechanism for initiating cardiovascular dysfunction, oxidative stress is usually increased in cigarette smoke exposure [16]. Furthermore, cigarette smoking impacts all phases of atherosclerosis from endothelial dysfunction to acute clinical events, and it is a powerful inducer of DNA methylation and gene expression alterations [17]. In addition, numerous toxicants from cigarette smoke perturb the dramatic balance of intestinal microbiota through various mechanisms. Fecal microbiome transplantation from mice previously exposed to cigarette smoke into germ-free mice naive to smoke exposure induces excessive weight gain across diets and mouse strains [18]. Cardiovascular diseases are closely associated with disorders of lipid metabolism in our bodies. Therefore, further investigation of dyslipidemia caused by smoking is important for the study of other smoking-induced diseases.

In the present study, cigarette smoke components (CSCs) were prepared and characterized, and SPF-grade SD rats were used to investigate the effect of CSCs on blood lipid levels, hepatic steatosis and gut microbiota. This study also investigated whether serum exosomes in rats affected lipid metabolism gene expression through miRNA and mRNA interactions.

## Materials and methods

### Instruments and materials

*An* Agilent 6890 N/5975 GC–MS gas chromatography-mass spectrometer (Agilent Technologies, California State, USA) with a DB-WAX capillary column (30 mm × 0.25 mm × 0.25 μm， Agilent Technologies, California State, USA) was used to analyze the chemical composition of the smoke gas. A high-performance liquid chromatography system (LC-20AT) and a prominent ultraviolet detector (Shimadzu Corporation, Japan) with an Agilent TC-C18 chromatographic column (5 μm, 150 mm × 4.6 mm， Agilent Technologies, California State, USA) were used to determine the nicotine content in the water-soluble components. The Agilent 1290 UPLC system (Agilent Technologies, California State, USA) was followed with a Q Exactive Focus high-resolution mass spectrometry system (Thermo Fisher Scientific, Massachusetts State, USA), which was used to detect the content of trimethylamine oxide (TMAO) in rat serum. A Spectra Max 190 full-wavelength microplate reader (Molecular Devices Corporation, California State, USA) was used to detect the biochemical indicators.

Filter cigarettes were made from single-grade tobacco leaves in our lab. The cigarette specification was (60 mm + 24 mm) × 24.9 mm, and the air permeabilities of the filter sponge and cigarette paper was 30.3 and 39%, respectively. The inspiratory resistance of the filter was 616 Pa, and the picadura weight of a single tobacco was 0.78 g. Serum indicators were detected by an ELISA kit purchased from Thermo Fisher Co., Ltd. Tumor necrosis factor (TNF-α), oxidized low-density lipoprotein (ox-LDL), and nuclear factor (NF-κB) ELISA kits were purchased from Beyotime Biotechnology Co., Ltd. HE staining and Rhodamine 123 standard were purchased from Aladdin (Shanghai) Biological Reagent Co., Ltd.

### Preparation of CSC and component analysis

**Fig. 1:**
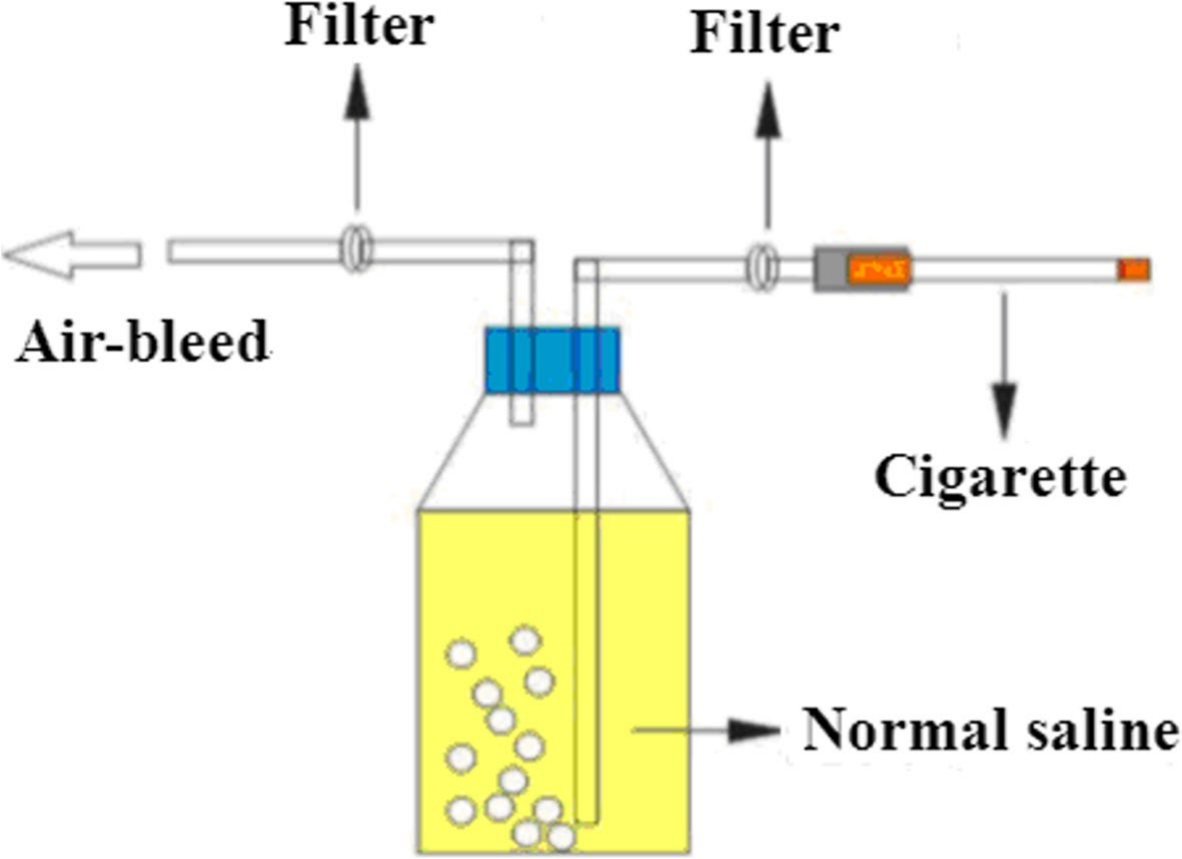
CSC preparation device. The cigarette was installed on a smoke generator, and the smoke was drawn into a screw-top bottle containing 100 mL of sterile physiological saline

According to the literature with slight modifications [8], the cigarette was installed on the smoke generator, which was a homemade flue gas component preparation device. The smoke was drawn into a screw-top bottle containing 100 mL of sterile physiological saline. Then, the air flow rate was regulated, and the draw time for one cigarette was approximately 4 minutes. Each 100 mL of sterile normal saline was filled with 8 cigarettes (0.08 cigarettes/mL). The smoke component preparation device is shown in Fig. 1.

After CSC was prepared, the obtained sample solution was filtered with a 0.45 μm ultramicrofiltration membrane and analyzed according to the GC–MS method with slight changes [9]. The CSC was separated into alkaline, neutral and acidic components. Briefly, (1) 10 mL CSC was extracted with 20 mL dichloromethane, vortexed, mixed for 2 min, and centrifuged at 5000 r/min for 10 min. The organic phase and aqueous phase were obtained. (2) The pH value of the above aqueous phase was adjusted to 1.0 with 20% sulfuric acid, and 20 mL dichloromethane was added, vortexed and mixed for 2 min and centrifuged at 5000 r/min for 10 min. The organic phase and aqueous phase were obtained. (3) The pH value of the above aqueous phase was adjusted to 13.0 with 20% NaOH, and 20 mL of dichloromethane was added. The aqueous phase was discarded, and the organic phase was washed with 10 mL of saturated NaCl solution and dried with 10 g of anhydrous sodium sulfate. After standing for an hour, all the organic phases were obtained and dried by a nitrogen stream in a 40 °C water bath, and 1 mL of dichloromethane was added to the residue and centrifuged at 15000 r/min for 10 minutes. Then, the neutral, acidic and alkaline components were obtained. In addition, 1.0 μL of the above-prepared analysis sample components were used for GC–MS analysis. Map data retrieval library: Willey library and NISTO2 library.

In addition, the RP-HPLC method was used to detect the nicotine content in the components. The mobile phase was acetonitrile-0.008 M potassium dihydrogen phosphate (containing 0.08 mmol/L heptane sulfonate, pH 3.0) =5–95 (v/v), flow rate was set at 0.8 mL/min, column temperature was set at 35 °C, UV detection wavelength 260 nm, injection volume was 20 μL.

### Animal experiments

SPF-grade SD male rats, weighing 125 ± 5 g, were purchased from Shanghai SLAC Laboratory Animal Co., Ltd., under laboratory animal license number SCXK (Shanghai) 2017–0005. These animal experiments were approved by the ethics committee of China Jiliang University. All rats were raised in a standard laboratory animal room (temperature 22 ~ 25 °C). In the experimental lab, the relative humidity was 65 ± 10%, and the 12 h/12 h cycle was day/night. All the rats were acclimatized in our laboratory for a week before the experiment. They were randomly divided into two groups: (A) the normal control group and (B) the CSC exposure group. The rats in the exposure group were orally exposed to CSC at 0.6 mg/kg (calculated as nicotine) for 6 weeks. Rat body weight was weighed each week, rat diet and water consumption per day were also recorded, and blood was collected every 2 weeks from the rat caudal vein to detect biochemical indexes. During the experiment, they were provided with granule feed and sterilized tap water ad libitum. The rat feed formula was composed of crude protein (≥ 20.5%), crude fat (≥ 4.0%), crude fiber (≥ 5.0%), crude ash (≤ 8.0%), calcium (1.0–1.8%), total phosphorus (0.6–1.2%), lysine (≥ 1.32%), methionine + cystine (≥ 0.78%), and sodium chloride (0.4%).

### Measurement of serum biochemical indicators

In strict accordance with the kit instructions, 12 biochemical indexes in rat serum were detected, including serum total cholesterol (TC), triglycerides (TGs), high-density lipoprotein cholesterol (HDL-C), low-density lipoprotein cholesterol (LDL-C), aspartate aminotransferase (AST), alanine aminotransferase (ALT), oxidized low-density lipoprotein (ox-LDL), catalase (CAT), superoxide dismutase (SOD), malondialdehyde (MDA), tumor necrosis factor (TNF-α) and nuclear factor (NF-κB). At the same time, the RP-HPLC or LC–MS/MS method was used to detect changes in the contents of free cholesterol (FC), ADP, ATP and trimethylamine oxide (TMAO) in the rat serum. In addition, the HPLC method was used to detect the contents of short-chain fatty acids (SCFAs) in rat feces. The specific method was slightly modified according to the literature [9–12]. Furthermore, the atherosclerosis index (AI) was also calculated.

Rat serum (0.2 mL) was transferred to a 10 mL tapered glass tube and vortexed for 1 min. Then, 0.2 mL ultrapure water and 1 mL n-hexane were added. Then, 0.6 mL ethanol was added to the above glass tube and vortexed for 2 min. After centrifugation (5000 r/min) for 10 min, all the organic phase (n-hexane) was transferred to a glass test tube. The organic phase was evaporated to dryness in a 45 °C water bath under a nitrogen stream. Then, 0.2 mL acetone was transferred to the dried tube, and 0.2 ml derivatization reagent (2 mol/L sulfuric acid and 2 mol/L chromium trioxide solution) was added. After oxidation for 1 h at room temperature, 0.6 mL ultrapure water and 1 mL n-hexane were added. After centrifugation (5000 r/min) for 10 min, all the organic phase (n-hexane) was transferred to a glass test tube. The organic phase was also evaporated to dryness. Then, 0.2 mL ethanol was transferred to the dried tube. After centrifugation (18,000 r/min) for 5 min, 20 μL was injected directly into the RP-HPLC system. The FC concentration in rat serum was determined.

A 0.1 mL serum sample was transferred into a 2 mL glass tube, and 0.4 mol/L perchloric acid solution (1:1) precooled at 4 °C was added. After vortexing for 2 min and centrifugation at 4 °C (15,000 r/min) for 10 min, all the supernatant was transferred into another glass tube, and then 10 μL of 2.0 mol/L potassium hydroxide solution precooled at 4 °C was added. After vortexing and centrifugation at 4 °C (15,000 r/min), all the supernatant was transferred and filtered with a 0.45 μm microporous membrane. Then, 20 μL was injected into the HPLC system for ATP, ADP and AMP analysis.

Serum (0.2 mL) was transferred into a 2 mL tube, and 0.2 mL ultrapure water was added. After shaking and mixing, 0.6 mL acetonitrile was added. After vortexing for 2 min and centrifugation at 18000 r/min for 10 min, all the supernatant was transferred into a 1.5 mL injection bottle, and 3.0 μL was injected into the LC–MS/MS system for TMAO analysis.

Rat cecal content (0.5 g) was transferred into a homogenizer, and 1 mL ultrapure water was added to homogenize the samples in an ice bath. After centrifugation at high speed (6000 r/min) and the addition of 0.1 mL of hydrochloric acid (HCl), 4 mL ether and 0.8 mL of sodium hydroxide were added to the homogenate. After vortexing, all the aqueous phases were collected and filtered with a 0.22 μm filter membrane, and 20 μL was injected into the HPLC for SCFA determination.

### Morphological observation of rat liver

After exposure to smoke components for 6 weeks, the rat was sacrificed, the liver was immediately removed, and the distal part of the left outer lobe of the liver was cut and fixed in 10% neutral formalin. After 24 h, they were embedded in liquid paraffin, sectioned at a thickness of 5 μm, and finally stained with hematoxylin & eosin (H&E).

Rat liver tissue (^≤1 mm3^) was fixed in 2.5% glutaraldehyde aqueous solution for more than 2 hours, rinsed twice with 0.1 M PBS, stained with 1% osmic acid solution for 1 hour, and rinsed with PBS and 2% uranyl acetate. Then, it was dehydrated with gradient alcohol. After treatment with alcohol and anhydrous acetone, the cells were observed under a transmission electron microscope (TEM).

### Mitochondrial permeability of liver cells

The specific evaluation method was described in the literature [14]. The fluorescent dye rhodamine 123 is used as a marker of mitochondrial membrane potential. The fluorescence intensity of the rat hepatocyte mitochondrial suspension in each group was measured by a fluorescence microplate reader at an excitation wavelength of 505 nm and an emission wavelength of 534 nm. The higher the fluorescence intensity was, the lower the mitochondrial membrane potential, which indicated greater membrane permeability of rat liver cell mitochondria.

### Gut microbial composition

After collecting the contents of the rat cecum, bacterial DNA was isolated by the QIAamp DNA Stool Mini Kit (QIAGNE, CA) according to the kit instructions. The concentration and purity of the extracted DNA were detected using a spectrophotometer. The V4 hypervariable region of the 16S rRNA gene was amplified with the primers 515F (5^/^−GTGCCAGCMGCCGCGGTAA-3^/^) and 806R (5^/^−GGACTACHVGGGTWTCTAAT-3^/^). The PCR was carried out by Phusion High-Fidelity PCR master Mix (New England Biolabs) under the following conditions: initial denaturation at 98 °C for 1 min, followed by 30 cycles of 10 s, annealing at 50 °C for 30 s, and elongation at 72 °C for 3 s, and a final extension at 72 °C for 30 s, and final extension at 72 °C for 5 min. PCR products were purified using a Gene JET Gel extraction kit (Thermo Scientific). Amplicons from different samples were mixed in equal amounts, which were sequenced by MKBio (Hangzhou, China) on an Illumina HiSeq 2500.

The sequencing data from our study in this paper were deposited in the NCBI Sequence Read Archive (SRA) associated with BioProject ID PRJNA761249 (https://www.ncbi.nlm.nih.gov/sra/PRJNA761249). Each operational classification unit OUT represents a DNA sequence with a sequence similarity greater than 97% and uses the Sliva and RPD databases to match the representative sequences of all OTUs to count each bacterial phylum in the sample.

### Real-time PCR

Total RNA from the liver tissues was extracted using a TRIzol® Plus RNA Purification Kit (Thermo Fisher, USA) according to the manufacturer’s protocol. SuperScript™ III First-Strand Synthesis SuperMix was used to synthesize cDNA (Thermo Fisher, USA) following the manufacturer’s instructions. GAPDH was selected as the reference gene, and Primer Premier 6.0 and Beacon Designer 7.8 were used to design quantitative PCR primers, which were synthesized by Shenggong Bioengineering Co., Ltd. (Shanghai, China). Real-time PCR was performed with PowerUp™ SYBR™ Green Master Mix (Applied Biosystems, USA) according to the manufacturer’s instructions. The primer sequences are shown in Table 1. The PCR conditions were as follows: 95 °C, 1 min; 40 cycles (95 °C, 15 s; 63 °C, 25 s; collecting fluorescence). The relative gene expression levels in different groups were statistically analyzed with the 2^-∆∆Ct^ method.

**Table 1:** RT-PCR primers for related genes in rat liver

Gene	Genbank Accession	Primer Sequences(5’to3’)	Size (bp)
Rat Prkaa2	NM_023991.1	GATGAGGCTGTGAAAGAAGTATGTG	157
		GGTAGAACTCACTGGCTTGGT	
Rat Acacb	NM_053922.1	GTCCTCGACTCCAGCATCAA	143
		GGGTCAATCCTCCTTATGGTCTT	
Rat Hmgcr	NM_013134.2	CCTGCGTGTCCCTGGTCCTA	125
		CCTTTGGGTTACTGGGTTTGGT	
Rat CYP7a1	NM_012942.2	CAAGACGCACCTCGCTATTCTCT	113
		CTTCAGAGGCTGCTTTCATTGCT	
Rat GAPDH	NM_017008.4	GAAGGTCGGTGTGAACGGATTTG	127
		CATGTAGACCATGTAGTTGAGGTCA	

### Western blot analysis

Approximately 20 mg of rat liver tissues was lysed in 150 μL of RIPA buffer. The total protein content of the samples was determined according to the instructions of the BCA kit. SDS–PAGE analysis was performed with 60 μg total protein in each lane, and then 2 h protein transfer was performed. After membrane transfer, the membrane was placed in T-TBS (containing 5% BSA) and sealed at room temperature for 1 h. The membranes were then incubated at 4 °C overnight with the following antibodies: rabbit anti-AMPK (1:500, Abcam ab3759), rabbit anti-p-AMPK (1:1000, CST2535), rabbit anti-ACC2 (1:2000, Abcam ab45174), rabbit anti-p-ACC2 (1:1500, CST11818), rabbit anti-HMG-CoAR (1:10000, Abcam ab174830), rabbit anti-p-HMG-CoAR (1:1000, Biorbyt orb6191), rabbit anti-CYP7A1 (1:1000, Biorbyt orb539102) and rabbit anti-GAPDH (Abcam ab181602, 1: 10000) as an internal control. Protein expression was visualized on X-ray films using a goat anti-mouse IgG secondary antibody (H + L) (Thermo Pierce, 1:5000) and goat anti-rabbit IgG secondary antibody (H + L) (Thermo Pierce, 1:5000) by SuperSignal® West Dura Extended Duration Substrate. ImageJ software was used to analyze the optical density values of the bands, and each band was repeated three times. The relative expression level of the target protein was represented by {target protein (optical density value)/internal reference (optical density value)} × 10^n^, and the results were expressed as the mean ± standard deviation.

### Peripheral serum exosomes

The blood samples were centrifuged at 2000×g for 30 min. The supernatant was separated, and rat serum was obtained. The supernatant was discarded after centrifugation at 10,0000×g for 120 min. Centrifugation was carried out at 4 °C. The exosome precipitate was resuspended in PBS, serum exosomes were obtained, and quantification was performed with a BCA kit from Thermo Fisher Co., Ltd. Exosomes were characterized through transmission electron microscopy (TEM), the average particle size of the exosomes was detected with a laser particle size analyzer, and exosome-labeled proteins CD9, CD63 and TSG101 were quantitatively detected through Western blotting.

### qRT–PCR assay for miRNA in serum exosomes

Approximately 300 μL of binding buffer was added to the exosomes, thoroughly shaken, and centrifuged at 12,000×g for 10 min. The supernatant was transferred to a spin cartridge, and the effluent was retained. Anhydrous ethanol was added to the effluent for the preparation of 70% ethanol, and the solution was thoroughly shaken. The mixture was transferred to a second spin cartridge and centrifuged at 12,000×g for 1 min. The waste liquid was discarded, and 500 μL of wash buffer was added to the spin cartridge. The sample was centrifuged at 12,000×g for 1 min, and the waste liquid was discarded. The previous step was repeated again. The adsorption column was dried and centrifuged again (12,000 g, 2 min). Finally, 50 μL of RNase-free ddH_2_O was added to the spin cartridge, stored at room temperature for 2 min, and stored at − 80 °C for subsequent use. qRT–PCR was performed according to the steps mentioned above. The reverse transcription primer sequences are shown in Table 2, and the qRT–PCR primers are shown in Table 3.

**Table 2:** Reverse transcription primer sequences of miRNAs

Gene	Genebank Accession	Reverse Transcription Primer Sequences(5′ to 3′)
*rno-mir-126a-5p*	MIMAT0000831	F: GTCGTATCCAGTGCAGGGTCCGAGGTATTCGCAC R: TGGATACGACCGCGTA
*rno-mir-291a-5p*	MIMAT0000895	F: GTCGTATCCAGTGCAGGGTCCGAGGTATTCGCAC R: TGGATACGACGGCACA
*Cel-miR-39-3p*	MIMAT0000010	F: GCTGTCAACGATACGCTACGTAACGGCATGACAG R: TGTTTTTTTTTTTTTCAAGCT

**Table 3:** Real-Time PCR Primers of miRNAs

Gene	Forward Primer and Universal Primer (5′ to 3′)
*rno-mir-126a-5p-F*	GCGCGCATTATTACTTTTGGTACG
*rno-mir-291a-5p-F*	GCGAAAGTGCTTCCACTTTGTG
*Cel-miR-39-3p-F*	CACCGGGTGTAAATCAGCTTG
*Universal reverse primer (micro-R)*	AGTGCAGGGTCCGAGGTATT

### Interaction of miRNAs on AMPK and CYP7A1 mRNA

According to bioinformatic analysis of miRNA binding sites, miRNA-291a-3p and miRNA-126a-5p from rat serum exosomes may be likely to bind or suppress AMPK and CYP7A1 expression. In this experiment, we wanted to determine whether miRNAs were affected by CSC exposure and participated in the posttranscriptional expression of AMPK and CYP7A1 mRNA. Consequently, the levels of miRNA-291a-3p and miRNA-126a-5p were determined by RT–PCR in the normal control group and CSC-exposed group. Subsequently, the interplay of miRNA and target mRNA was assessed and validated by a dual luciferase reporter gene assay (DLRG). A commercially available pmirGLO vector (Promega, Madison, USA) was reconstructed with wild-type and mutant 3′-UTR fragments of AMPK or CYP7A1 mRNA and containing the putative miRNA binding sequence. Luciferase activity assays were performed and normalized relative to *Renilla* luciferase activity.

### Statistical analyses

SPSS 11.5 statistical software was used for one-way analysis of variance (one-way ANOVA), and the data were expressed as^−^x ± s after statistical analysis. *P < 0.05* indicates a significant difference.

## Results

### GC–MS analysis of CSCs

Some chemicals in CSC were detected and identified by GC–MS, including 16 neutral components (Table 4), 44 acidic components (Table 5), and 40 alkaline components (Table 6). Among them, the compounds with the highest relative content were glycerol triacetate, 2,4-(1,1-dimethylethyl) phenol and nicotine, which accounted for 75.92, 17.63, and 39.29% of all detected components, respectively.

**Table 4:** Chemical composition and relative content of neutral components

No.	Molecular formula	chemical name	Relative content(%)
1	C_2_Cl_6_	Ethane, hexachloro-	0.838
2	C_13_H_28_	Decane, 2,3,7-trimethyl-	0.333
3	C_9_H_14_O_6_	Triacetin	75.920
4	C_20_H_42_	Eicosane	2.989
5	C_14_H_27_O	Phenol, 2,4-bis(1,1-dimethylethyl)-	5.193
6	C_14_H_10_	Diphenylethyne	0.805
7	C_9_H_7_O_2_	4-Vinylbenzoic acid	1.267
8	C_18_H_38_	5,5-Dibutylnonane	1.207
9	C_25_H_52_	Pentacosane	0.587
10	C_21_H_44_	Heneicosane	0.535
11	C_24_H_50_	Tetracosane	1.537
12	C_17_H_34_O_2_	Hexadecanoic acid, methyl ester	5.976
15	C_28_H_58_	Octacosane	1.438
16	C_18_H_38_	Octadecane	1.376

**Table 5:** Chemical composition and relative content of acidic components

No.	Molecular formula	chemical name	Relative content(%)
1	C_13_H_28_	Decane, 2,4,6-trimethyl	1.629
2	C_2_Cl_6_	Ethane, hexachloro	2.831
3	C_13_H_28_	Tridecane	0.444
4	C_9_H_20_O_2_Si	Silane, cyclohexyl dimethoxy methyl	0.821
5	C_13_H_22_O_2_	E-2-Octenyl tiglate	0.459
6	C_15_H_32_	Tetradecane, 5-methyl	0.773
7	C_4_Cl_6_	1,3-Butadiene, 1,1,2,3,4,4-hexachloro	0.770
8	C_13_H_28_	Undecane, 4,4-dimethyl	0.801
9	C_11_H_24_	Octane, 5-ethyl-2-methyl	1.106
10	C_43_H_88_	Tritetracontane	0.252
11	C_10_H_16_O	3-Cyclopentene-1-acetaldehyde, 2,2,3-trimethyl	0.599
12	C_16_H_34_	Hexadecane	2.614
13	C_21_H_44_	Eicosane, 10-methyl	0.796
14	C_10_H_21_Br	Decane, 3-bromo	0.722
15	C_26_H_54_	Hexacosane	1.048
16	C_21_H_44_	Heneicosane	4.200
17	C_22_H_46_	Docosane	3.323
18	C_17_H_36_	Tetradecane, 2,6,10-trimethyl	0.546
19	C_14_H_22_O	Phenol, 2,4-bis(1,1-dimethylethyl)	17.634
20	C_17_H_36_	Heptadecane	3.319
21	C_24_H_50_	Tetracosane	1.574
22	C_18_H_37_Cl_3_Si	Silane, trichlorooctadecyl	0.782
23	C_20_H_42_	Eicosane	5.412
24	C_13_H_28_	Nonane, 5-butyl	0.721
25	C_30_H_62_	Triacontane	3.092
26	C_25_H_52_	Pentacosane	3.082
27	C_34_H_70_	Tetratriacontane	3.830
28	C_26_H_54_	Heneicosane, 11-(1-ethylpropyl)	0.890
29	C_16_H_34_	Pentadecane, 3-methyl	0.520
30	C_18_H_38_	Pentadecane, 2,6,10-trimethyl	1.339
31	C_17_H_36_	Hexadecane, 2-methyl	2.223
32	C_25_H_52_	Heptadecane, 9-octyl	0.901
33	C_25_H_52_	Pentacosane	2.965
34	C_27_H_56_	Heptacosane	9.603
35	C_17_H_24_O_3_	7,9-Di-tert-butyl-1-oxaspiro (4,5) deca-6,9-diene-2,8-dione	2.166
36	C_21_H_34_O_3_	Benzenepropanoic acid, 3,5-bis(1,1-dimethylethyl)-4-hydroxy-, methyl ester	0.982
37	C_28_H_58_	Octacosane	5.867
38	C_31_H_64_	Hentriacontane	2.137
39	C_16_H_34_O	Tridecanol, 2-ethyl-2-methyl	1.416
40	C_10_H_18_Si_2_	1,4-Bis (trimethylsilyl)-1,3-butadiyne	0.348
41	C_18_H_38_	Octadecane	0.919
42	C_18_H_20_	9H-Fluorene, 9-butyl-9-methyl	1.808
43	C_11_H_11_NOS	Benz [b]-1,4-oxazepine-4(5H)-thione,2,3-dihydro-2,8-dimethyl	1.452
44	C_16_H_16_	Benzene, 1,1′-(2-butene-1,4-diyl) bis-	1.282

**Table 6:** Chemical composition and relative content of alkaline components in CSC

No.	Molecular formula	chemical name	Relative content(%)
1	C_12_H_26_	Undecane, 5-methyl-	0.953
2	C_6_H_4_F_2_	2,3-Difluorophenol	2.908
3	C_2_Cl_6_	Ethane, hexachloro-	0.834
4	C_7_H_11_NO	1-Azabicyclo [2.2.2] octan-3-one	7.017
5	C_9_H_20_O_2_Si	Silane, cyclohexyldimethoxymethyl-	1.311
6	C_11_H_24_	Octane, 2,4,6-trimethyl-	0.312
7	C_13_H_28_	Undecane, 4,4-dimethyl-	0.569
8	C_11_H_24_	Decane, 2-methyl-	0.288
9	C_9_H_14_O_6_	Triacetin	2.020
10	C_10_H_14_N_2_	Nicotine	39.287
11	C_15_H_32_	Tetradecane, 5-methyl-	0.834
12	C_21_H_44_	Heneicosane	1.760
13	C_21_H_44_	Heptadecane, 2,6,10,15-tetramethyl-	0.307
14	C_16_H_34_	Hexadecane	0.568
15	C_22_H_42_O_4_	Oxalic acid, bis(6-ethyloct-3-yl) ester	0.266
16	C_17_H_36_	Tetradecane, 2,6,10-trimethyl-	0.955
17	C_28_H_58_	Octacosane	2.168
18	C_17_H_36_	Hexadecane, 3-methyl-	0.392
19	C_12_H_26_	Decane, 3,8-dimethyl-	1.468
20	C_14_H_22_O	Phenol, 2,4-bis(1,1-dimethylethyl)-	10.395
21	C_12_H_25_Br	2-Bromo dodecane	0.679
22	C_13_H_28_	Tridecane	0.494
23	C_20_H_42_	Eicosane	2.621
24	C_24_H_50_	Tetracosane	0.398
25	C_13_H_28_	Nonane, 5-butyl-	0.589
26	C_15_H_32_	Pentadecane	0.744
27	C_17_H_36_	Hexadecane, 2-methyl-	1.840
28	C_18_H_38_	Octadecane	1.907
29	C_18_H_37_I	Octadecane, 1-iodo-	0.692
30	C_25_H_52_	Pentacosane	2.105
31	C_17_H_36_	Heptadecane	0.428
32	C_13_H_28_	Decane, 2,3,5-trimethyl-	2.092
33	C_17_H_24_O_3_	7,9-Di-tert-butyl-1-oxaspiro (4,5) deca-6,9-diene-2,8-dione	1.162
34	C_18_H_28_O_3_	Benzenepropanoic acid, 3,5-bis (1,1-dimethylethyl)-4-hydroxy-, methyl ester	1.262
35	C_27_H_56_	Heptacosane	4.690
36	C_34_H_70_	Tetratriacontane	0.580
37	C_22_H_46_	Docosane	0.789
38	C_18_H_21_O	9-Octadecenamide, (Z)-	0.672
39	C_4_H_4_N_3_Cl	4-Chloro-6-aminopyrimidine	2.931
40	C_18_H_38_	Heptadecane, 3-methyl-	0.526

### Physiological indexes of rats were markedly disturbed by CSC exposure

**Fig. 2:**
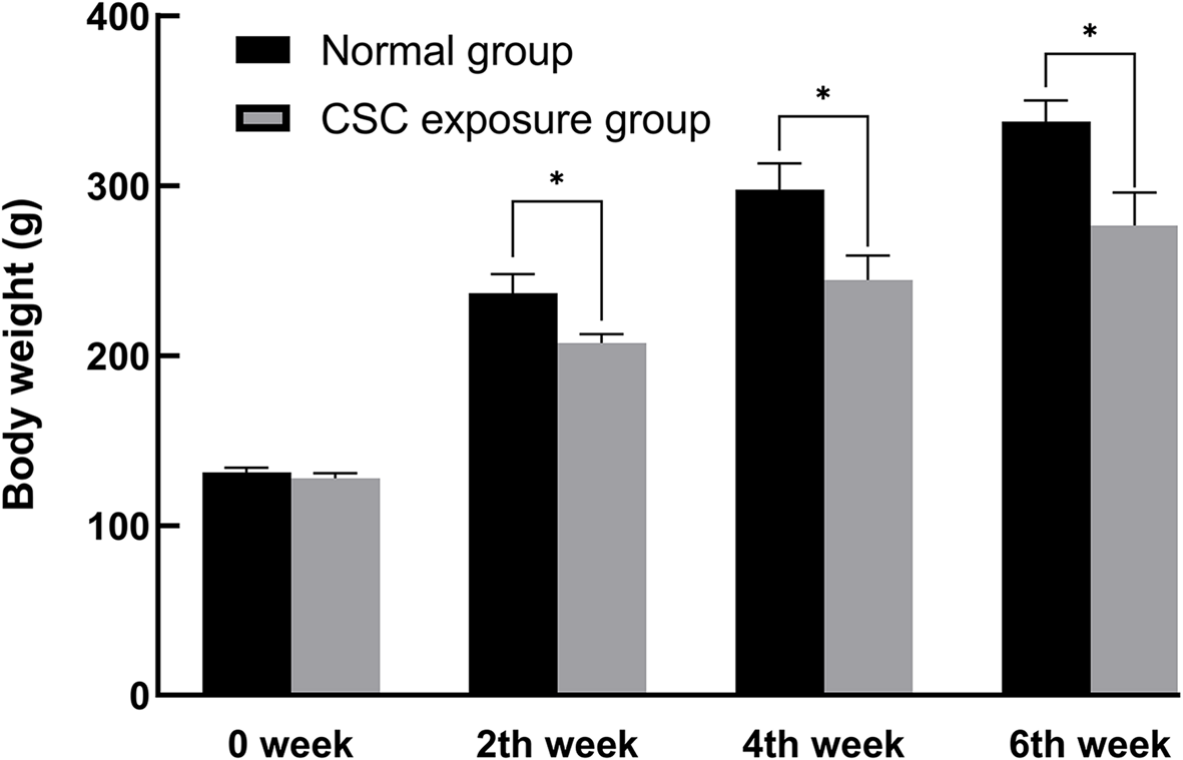
The effect of CSC exposure on the body weight of rats. Black columns represent the body weight after normal diet treatment, and the gray columns represent the body weight after oral administration of CSC. Compared with the normal group, the rat body weight was significantly reduced after treatment with CSC. A significant difference is indicated with the sign _*_ (*p* < 0.05)

As shown in Fig. 2, rat weights were increased over 6 weeks in the normal control group and CSC exposure group. Compared with the normal control group, body weight gained slightly slower in the CSC exposure group (*P* < 0.05). Diet and water consumption exhibited no significant difference between these two groups.

In Table 7, compared with the normal group, the proportion of serum T-CHO in the CSC group was significantly increased at the 2nd week and 4th week (*P* < 0.05), and there was no significant difference in the other indicators in serum. After 4 weeks of CSC exposure, serum T-CHO, LDL-C, AST/ALT and AI of rats in the CSC group were further increased, and the levels of TG and HDL-C decreased. At the end of 6 weeks, compared with the normal group, the trends of serum T-CHO, LDL-C, AST/ALT and AI in the CSC group remained significantly increased (*P* < 0.05), while the levels of TG and HDL-C were significantly decreased (*P* < 0.05).

**Table 7:** Levels of serum lipids and liver function in rats after CSC exposure (*n* = 6)

Period	Group	T-CHO mmol/L	TG mmol/L	HDL-C mmol/L	LDL-C mmol/L	AST/ALT	AI (%)
2th week	NC	2.49 ± 0.31	1.64 ± 0.47	1.05 ± 0.44	1.04 ± 0.22	1.69 ± 0.37	1.77 ± 0.93
	CSC	2.99 ± 0.24*	1.11 ± 0.45	0.99 ± 0.18	1.27 ± 0.16	2.32 ± 0.17	2.18 ± 0.85
4th week	NC	2.53 ± 0.32	1.62 ± 0.41	1.08 ± 0.08	1.09 ± 0.14	1.68 ± 0.14	1.35 ± 0.37
	CSC	3.21 ± 0.21	0.93 ± 0.31	0.92 ± 0.07	1.69 ± 0.27*	2.62 ± 0.25*	2.54 ± 0.49*
6th week	NC	2.51 ± 0.08	1.65 ± 0.24	1.09 ± 0.07	1.09 ± 0.07	1.57 ± 0.17	1.32 ± 0.11
	CSC	3.14 ± 0.14*	0.92 ± 0.27*	0.89 ± 0.06*	1.63 ± 0.09*	2.95 ± 0.27*	2.52 ± 0.13*

Compared with the normal group (NC), *P < 0.05, the serum atherogenic index AI = (TC-HDL)/HDL

**Fig. 3:**
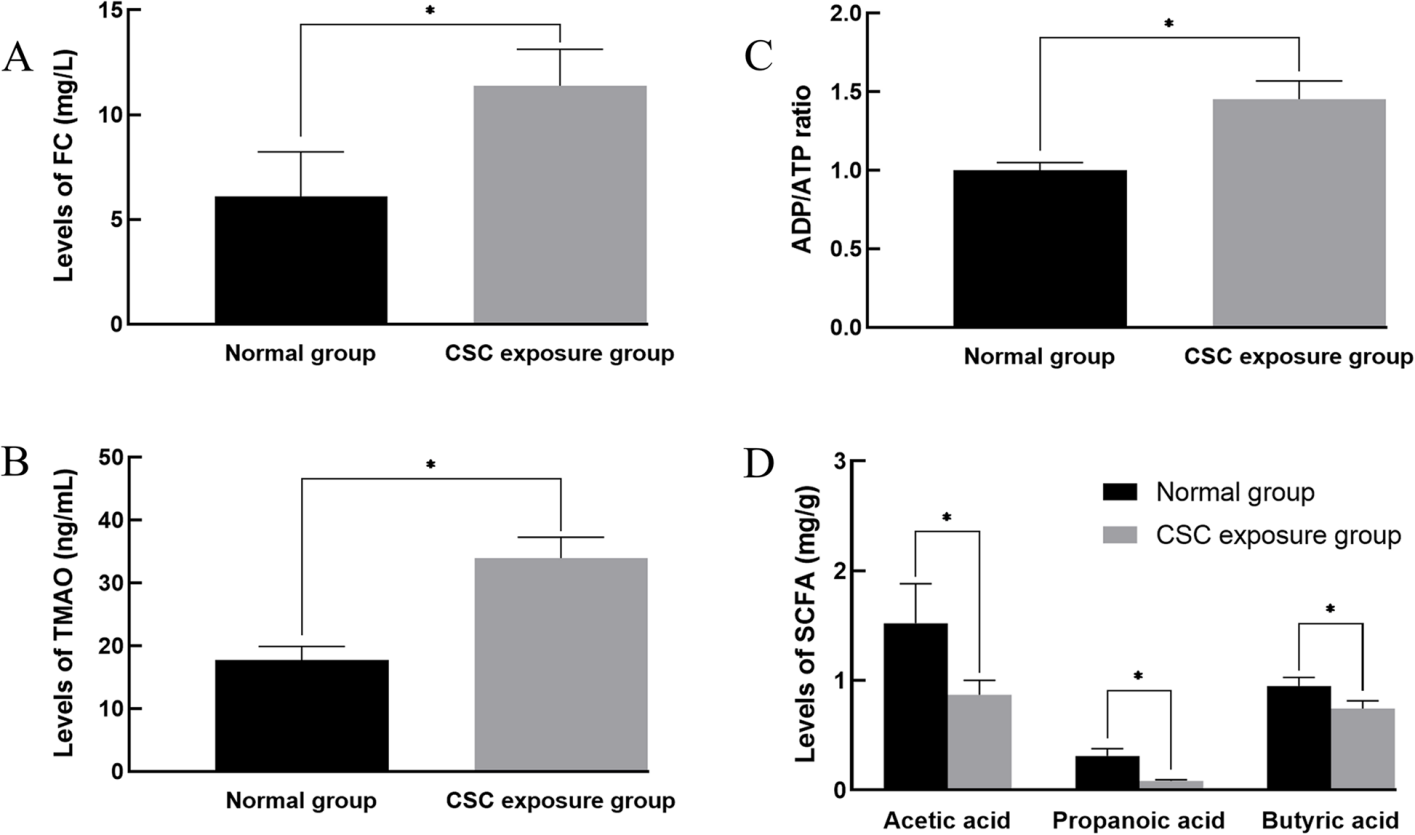
Indexes of rat serum in different treatment groups. In **A**, **B** and **C**, the contents of free cholesterol, ADP, ATP and trimethylamine oxide in the rat serum were significantly increased. In **D**, the contents of acetic acid, propropanoic acid and butyric acid in rat feces were significantly decreased. A significant difference is indicated with the sign _*_ (*p* < 0.05)

The RP-HPLC and LC–MS/MS methods were used to detect the levels of free cholesterol, ADP, ATP and TMAO in rat serum, which are shown in Fig. 3. After 6 weeks of CSC exposure, the FC concentration in rat serum (Fig. 3A) was significantly increased in comparison with the normal control group (*P* < 0.05). The ratio of ADP/ATP (Fig. 3B) and the level of trimethylamine oxide (Fig. 3C) in the CSC exposure group were also significantly increased (*P* < 0.05). However, compared with the normal control group, the SCFA (acetic acid, propionic acid and butyric acid) contents (Fig. 3D) in rat feces from the CSC exposure group were significantly decreased (*P* < 0.05).

### Effects of CSC exposure on oxidative stress in rats

By measuring the six indicators CAT, MDA, SOD, NF-κB, TNF-α and ox-LDL in rat serum during CSC exposure, it can be seen from Table 8 and Table 9 that the levels of serum CAT and NF-κB in the CSC exposure group were significantly increased at the 4th week and 6th week (*P* < 0.05), while the levels of MDA and SOD were decreased. The levels of serum TNF-α and ox-LDL in the CSC exposure group were not significantly different from those in the normal control group.

**Table 8:** Changes of CAT, MDA and SOD in rat after CSC exposure (*n* = 6)

Period	Group	CAT(U/mL)	MDA (nmol/mL)	SOD(U/mL)
4th week	NC	1.66 ± 0.27	19.85 ± 3.21	22.92 ± 0.81
	CSC	2.40 ± 0.14*	14.96 ± 2.91*	20.03 ± 0.98*
6th week	NC	1.63 ± 0.21	19.87 ± 1.38	23.39 ± 0.49
	CSC	2.33 ± 0.22*	11.57 ± 3.36*	19.58 ± 0.64*

Compared with NC group, *P < 0.05

**Table 9:** Changes of NF-κB, TNF-α and ox-LDL in rat after CSC exposure (*n* = 6)

Period	Group	NF-κB (pg/mL)	TNF-α (pg/mL)	ox-LDL (pg/mL)
4th week	NC	104.96 ± 14.87	98.21 ± 9.46	1.52 ± 0.26
	CSC	134.31 ± 7.48*	91.86 ± 9.54	1.66 ± 0.21
6th week	NC	106.62 ± 10.71	92.15 ± 7.08	1.68 ± 0.15
	CSC	139.55 ± 5.63*	98.48 ± 5.39	1.56 ± 0.24

Compared with NC group, *P < 0.05

### Effect of CSC exposure on the morphology of rat liver

To further verify the toxicity of CSC exposure, the organ coefficient and fat coefficient were calculated after the rats were sacrificed, and H&E staining and TEM detection were performed to observe pathological changes in the liver. Table 10 shows that the liver organ coefficient and renal organ coefficient of rats in the CSC exposure group were increased significantly (*P* < 0.05), and the fat coefficient decreased significantly in comparison with the normal control group (*P* < 0.05).

**Table 10:** Effects of CSC on organ coefficient and fat coefficient (%) of rats (*n* = 6)

Group	liver		kidney		fat	
	Weight (g)	Organ coefficient	Weight (g)	Organ coefficient	Weight (g)	Organ coefficient
NC	10.52 ± 1.05	3.08 ± 0.03	2.16 ± 0.23	0.63 ± 0.01	5.61 ± 0.62	1.64 ± 0.03
CSC	9.44 ± 0.55	3.22 ± 0.03*	2.57 ± 0.19*	0.87 ± 0.04*	4.17 ± 0.08*	1.42 ± 0.06*

Compared with NC group, *P < 0.05

**Fig. 4:**
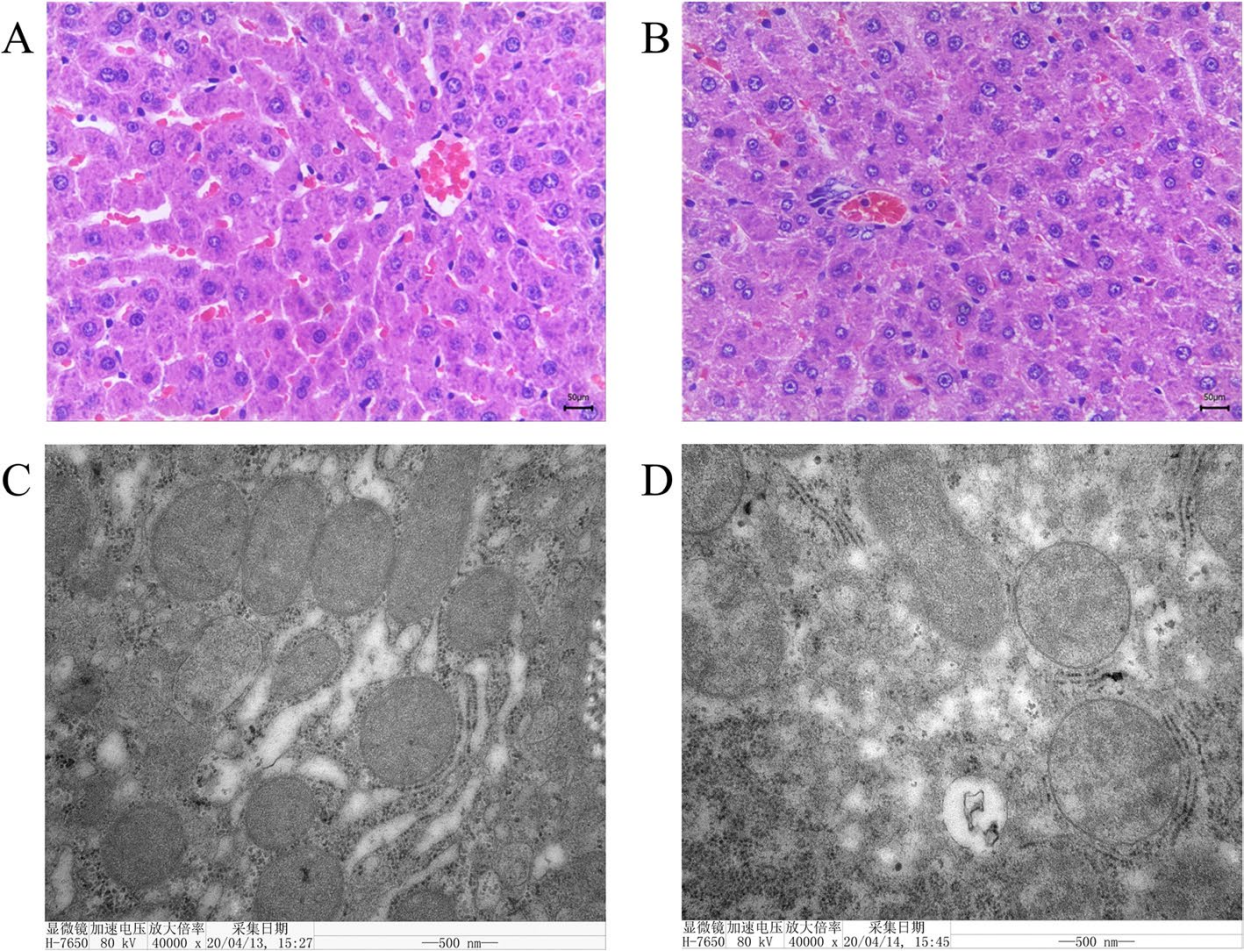
HE staining and transmission electron microscope observation in rat liver. **A** HE staining of liver tissue sections from normal group rats showed normal liver histology. **B** Liver HE staining from CSC group rats treated with normal diet and oral CSC. The hepatic sinusoid was slightly irregular, and scattered fat droplets were also observed in hepatocytes. **C** Hepatocytes in the normal control group were relatively complete. **D** The subcellular structure in the CSC exposure group was disrupted, including the mitochondrial and endoplasmic reticulum membranes

The results of HE staining of rat liver are shown in Fig. 4. The liver lobules and liver cells of the normal control group had clear structures and were arranged in ropes (Fig. 4A). The liver cells in the CSC exposure group showed little steatosis, unclear liver lobes and disordered arrangement of hepatic cords. The hepatic sinusoid was slightly irregular, and scattered fat drops were also observed in hepatocytes (Fig. 4B).

Transmission electron microscopy (TEM) was used to observe liver tissue changes. Hepatocytes in the normal control group were relatively complete, and the inner and outer membrane boundaries were clear (Fig. 4C), while the subcellular structure in the CSC exposure group was disrupted, including mitochondrial and endoplasmic reticulum membranes (Fig. 4D).

### CSC exposure markedly enhanced the mitochondrial permeability of hepatocytes

**Fig. 5:**
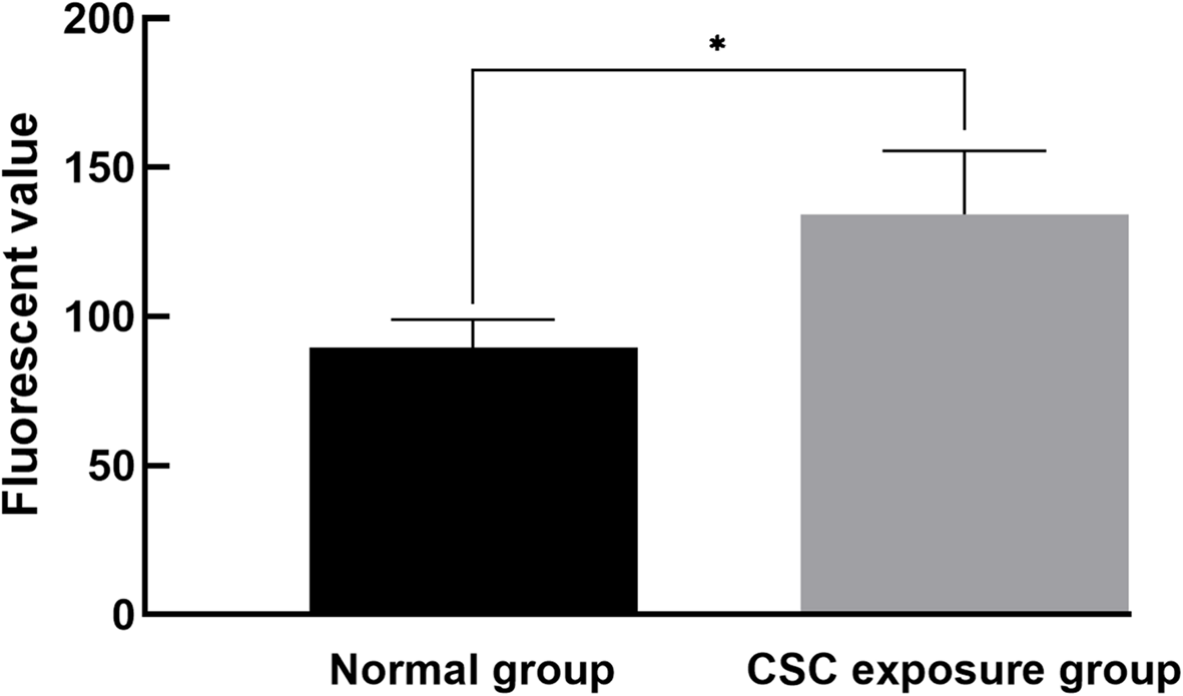
Changes in mitochondrial membrane potential in rat hepatocytes. Black columns represent the fluorescence intensity in the normal control group, and gray columns represent the fluorescence intensity in the CSC exposure group. There was significant damage to the permeability of rat hepatocyte mitochondria induced by CSC. A significant difference is indicated with the sign _*_ (*p* < 0.05)

Using a fluorescence microplate reader, the fluorescence intensity of the rat hepatocyte mitochondrial suspension in the normal control group was 89.50 ± 9.45, while the value in the CSC exposure group was 134.25 ± 21.33, as shown in Fig. 5. After statistical analysis, there was a significant difference in the mitochondrial membrane potential between the normal control group and the CSC exposure group (*P* < 0.05), suggesting that there was significant damage to the permeability of rat hepatocyte mitochondria induced by CSCs.

### CSC exposure significantly disturbed the gut microbial community in rats

**Fig. 6:**
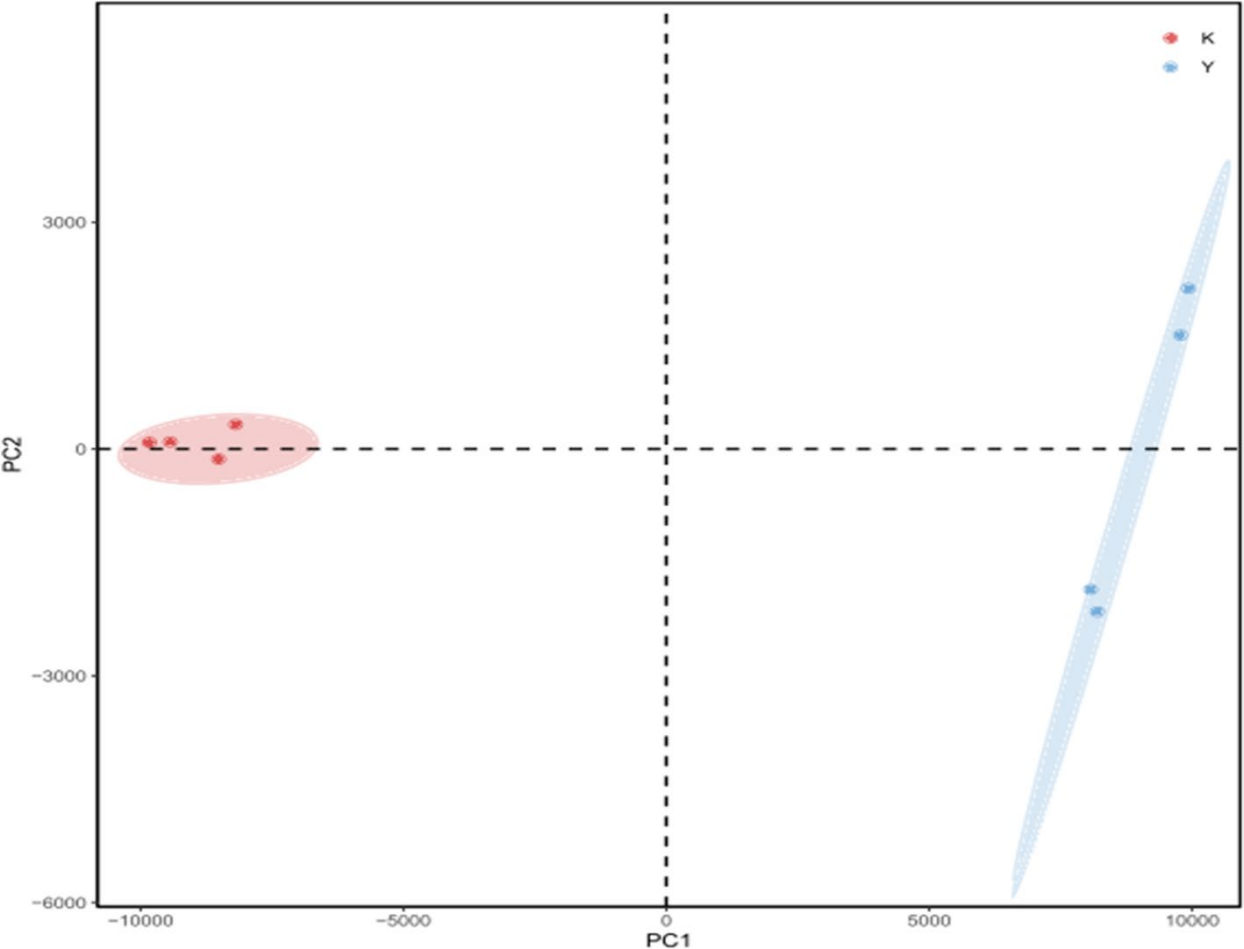
Biogeography of the gut microbiome between the NC group and CSC exposure group. The red dots represent the intestinal flora of the normal group, and the blue dots represent the CSC exposure group. There was a significant difference in the bacterial community between the normal control group and the CSC exposure group

To study the effect of CSC exposure on the structural community of intestinal flora in rats, we sequenced the V4 regions of the 16S RNA gene of the cecal microbes and analyzed the bacterial community at the phylum and genus levels. There was a significant difference in the bacterial community between the normal control group and the CSC exposure group, which is shown in Fig. 6.

**Fig. 7:**
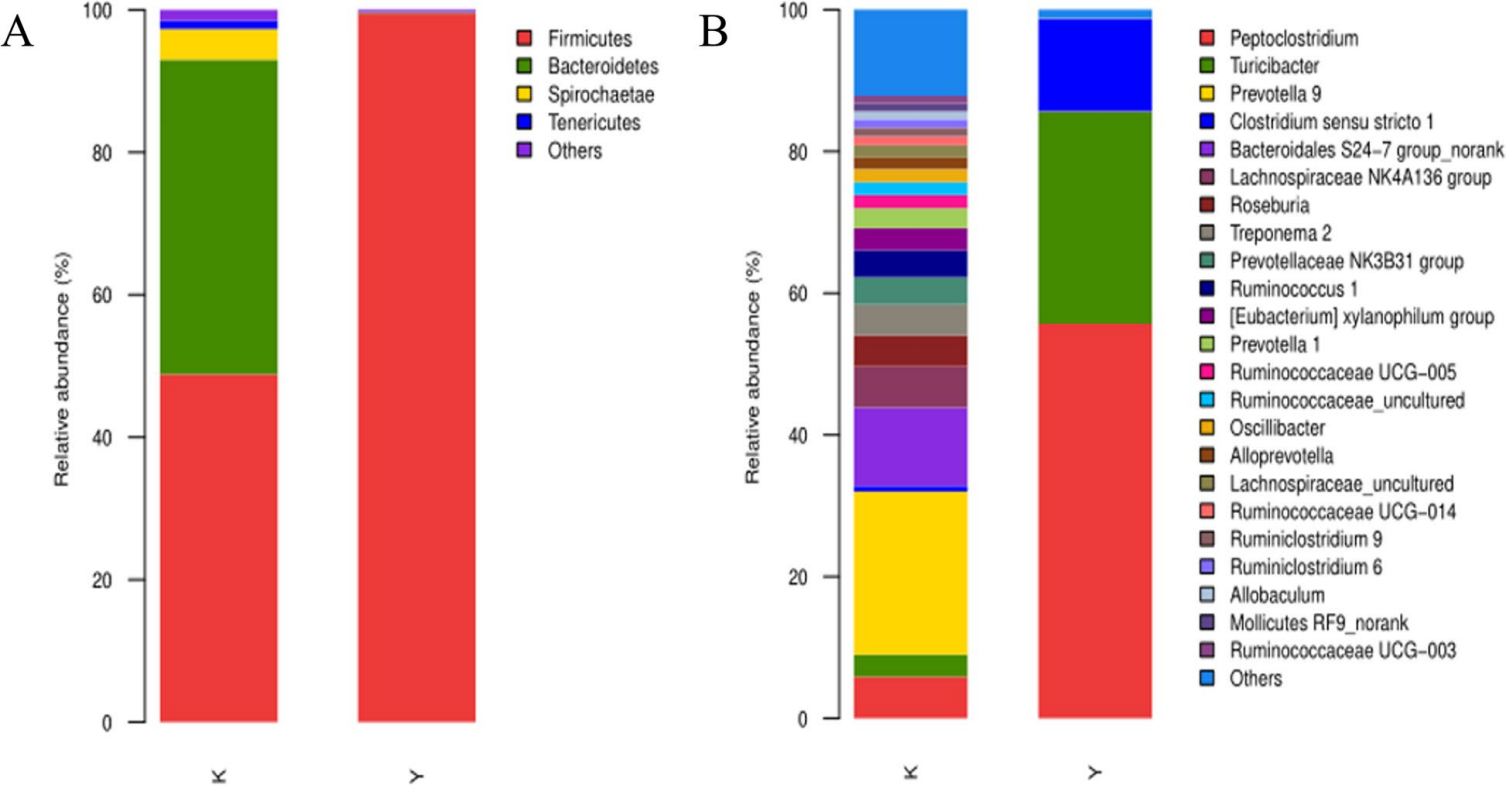
Community composition of gut microbiota in the NC group and CSC exposure group at the phylum and genus levels, respectively. **A** The gut microbiota at the phylum level. **B** The gut microbiota at the genus level. (K) NC group. (Y) CSC exposure group. At the phylum level, the bacterial taxa varied greatly between the normal control group and the CSC exposure group. At the genus level, Prevotella 9 and Bacteroidales were the two predominant genera in the normal control group. Peptoclostridium, Turicibacter and Clostridium sensu stricto were the most abundant genera in the CSC exposure group

At the phylum level, the bacterial taxa varied greatly between the normal control group and the CSC exposure group. Among these taxa, *Firmicutes* was the most predominant in both the normal control group and CSC exposure group, representing > 50 and > 95% in the normal control group and CSC exposure group, respectively. *Bacteroidetes* constituted the second most abundant phylum, and *Spirochaetota* ranked third in the normal control group. The other phyla were relatively minor, accounting for < 10% of the bacterial population in both the normal control group and CSC exposure group (Fig. 7A). At the genus level, *Prevotella 9* and *Bacteroidales* were the two predominant genera, accounting for > 40% of all bacteria in the normal control group. However, *Peptoclostridium*, *Turicibacter* and *Clostridium* sensu stricto were the most abundant genera, accounting for > 95% of all bacteria in the CSC exposure group (Fig. 7B).

**Fig. 8:**
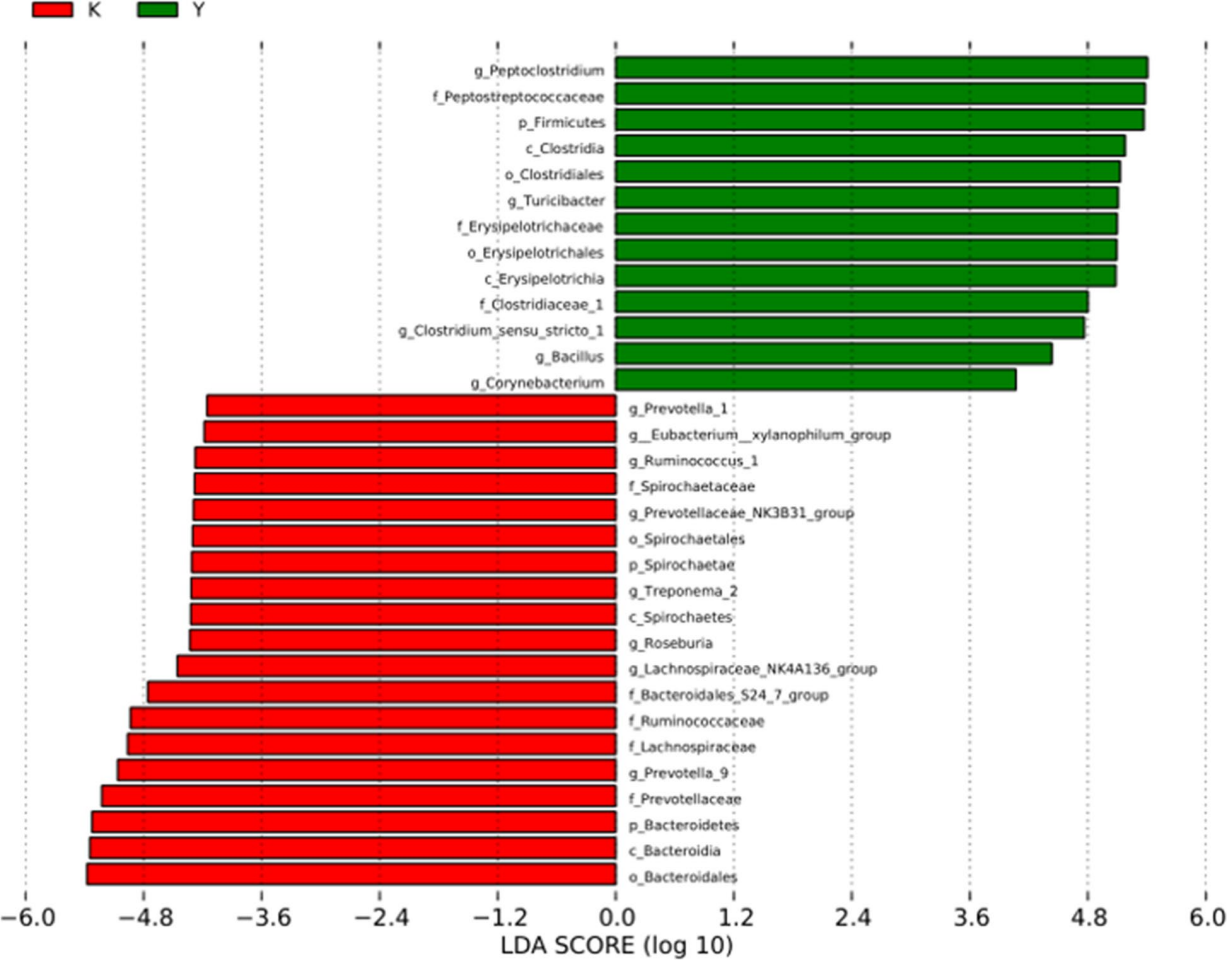
Bacterial taxa differentially represented in the NC group and CSC exposure group by LEFSe using an LDA score threshold of > 3.0. (K) NC group (Y) CSC exposure group. An LDA score threshold greater than 3.0 shows a significant difference in the NC group and CSC exposure group

To identify enrichment of certain bacterial taxa between the NC group and CSC exposure group, LEfSe was developed, which emphasized both significant differences and biological consistency. Based on the logarithmic LDA score of 3.0 as the threshold value, many OTUs were significantly exhibited between the NC and CSC exposure groups. Some were uniquely enriched in the NC and CSC exposure groups, and a number of OTUs were more prevalent (Fig. 8).

### CSC exposure significantly interfered with gene expression in the rat liver at the mRNA and protein intensity levels

To further explore the mechanism of smoke exposure on blood lipid metabolism in rats, we used qRT–PCR and Western blotting to detect the expression levels of lipid metabolism-related genes in the liver.

**Fig. 9:**
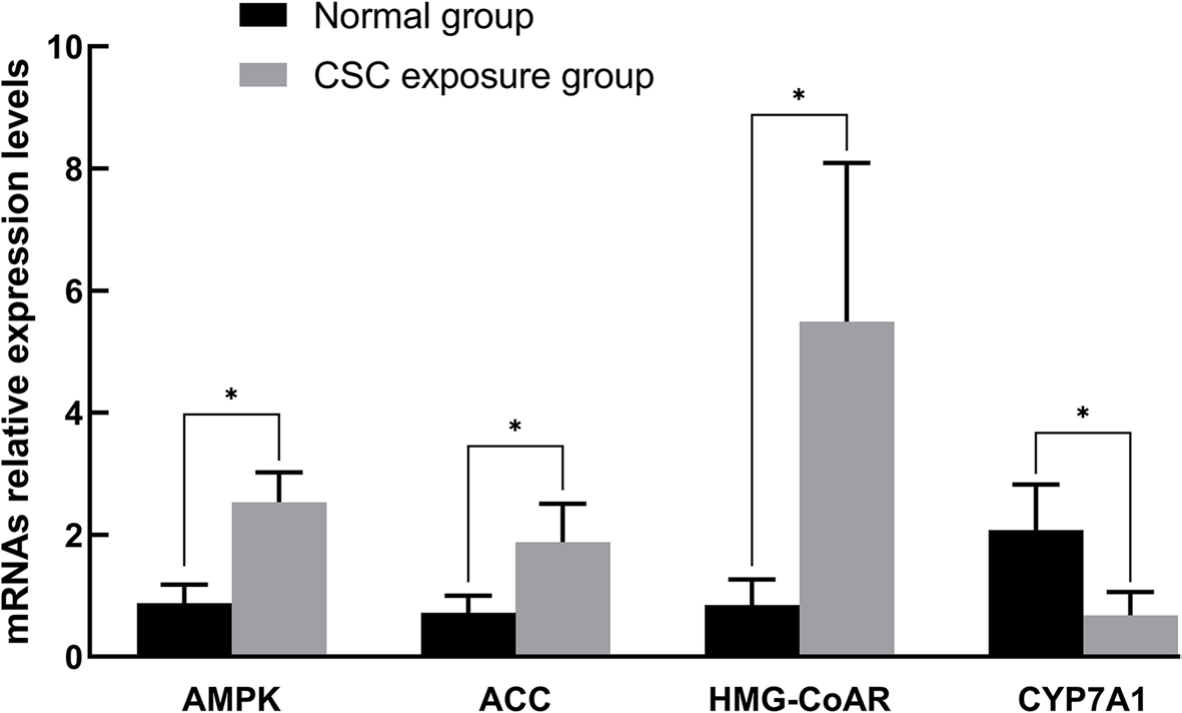
The RT–PCR levels of liver genes in the NC and CSC exposure groups. Total RNA was extracted from liver tissue, and the expression levels of related genes in the liver were detected by RT–PCR. A significant difference is indicated with the sign _*_ (*p* < 0.05)

As shown in Fig. 9, compared with the normal control group, the expression levels of Prakaa2 (AMP-activated protein kinase-α2, AMPK-α2) and Acacb2 (acetyl-CoA carboxylase 2, ACC2) in the CSC exposure group were significantly increased (*P* < 0.05), and the Hmgcr (hydroxymethylglutaryl-CoA reductase, HMG-CoR) expression level was also increased significantly (*P* < 0.05). However, the expression level of CYP7A1 (cholesterol 7α dehydrogenase) was significantly reduced (*P* < 0.01) in the CSC exposure group.

**Fig. 10:**
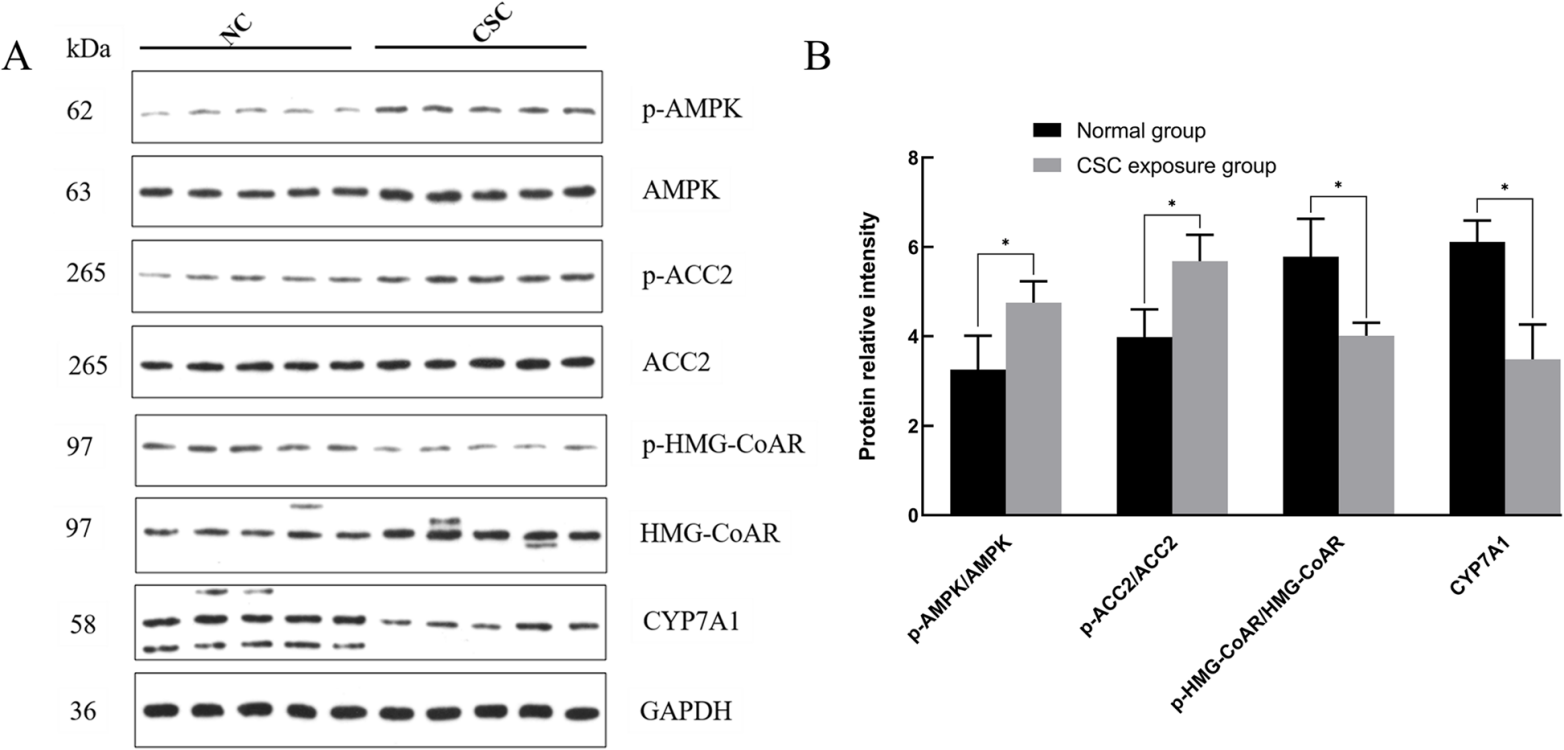
Western blotting levels of liver proteins in the NC and CSC exposure groups. The western blotting results showed that the p-AMPK/AMPK and p-ACC2/ACC2 protein expression levels in the CSC exposure group were significantly increased, and the expression levels of p-HMG-CoAR/HMG-CoAR and CYP7A1 protein were significantly decreased. A significant difference is indicated with the sign _*_ (*p* < 0.05)

As shown in Fig. 10, compared with those in the normal control group, the p-AMPK/AMPK and p-ACC2/ACC2 protein expression levels in the CSC exposure group were increased significantly (*P* < 0.05), while the protein expression levels of p-HMG-CoAR/HMG-CoAR and CYP7A1 were significantly decreased (*P* < 0.05).

### Identification and characterization of serum exosomes

**Fig. 11:**
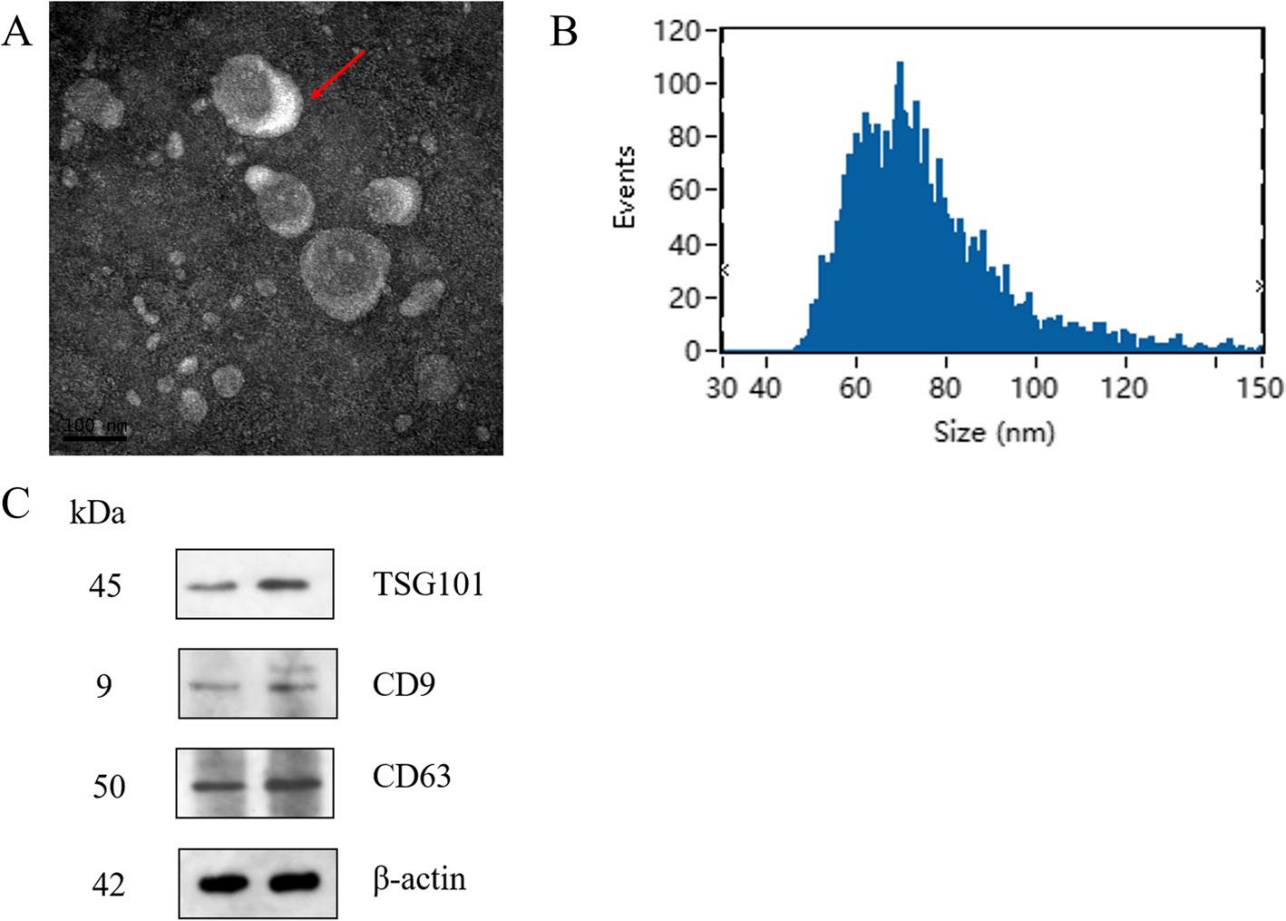
Morphology and identification of exosomes. **A** Serum exosomes from different groups were observed by transmission electron microscopy (TEM) at a scale of 100 nm and are indicated with arrowheads. **B** Particle size analysis showed that the particle size of each exosome ranged from 40 nm to 100 nm. **C** The expression levels of TSG101, CD9 and CD63 were determined by western blot. These proteins were extracted from exosomes isolated from rat serum and were always used as the labeled proteins in exosomes

TEM, dynamic light scatter size determination and Western blotting were used to characterize the isolated serum exosomes. TEM observation showed that the serum exosomes showed spherical structures with different sizes, as shown in Fig. 11A. Particle size analysis showed that the particle size range of the exosomes was 40–100 nm (Fig. 11B), and the average particle sizes of the NC and CSC exposure groups were 78.33 and 74.69 nm, respectively. Western blotting was used to analyze the surface label proteins of exosomes, such as CD63, CD9 and TSG101 (Fig. 11C), and the results showed that the expression levels of marker proteins were high in the exosomes.

### miRNA in serum exosomes markedly regulated the expression of AMPK and CYP7A1

**Fig. 12:**
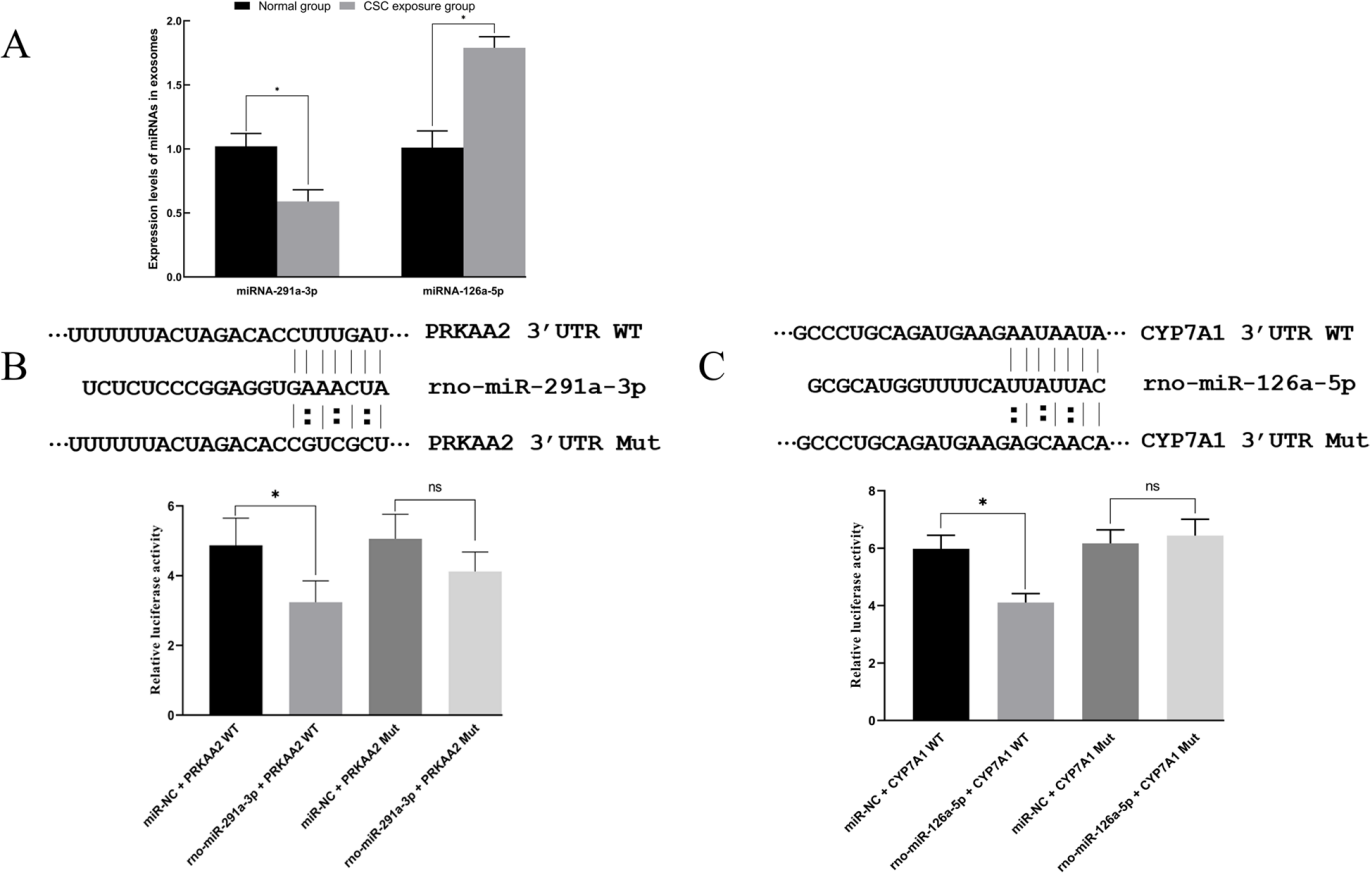
Effect of miRNA-291a-3p and 126a-5p on AMPK and CYP7A1 mRNA. (**A**) The expression levels of miRNA-291a-3p and 126a-5p in rat serum. The interplay of miRNA-291a-3p with AMPK mRNA was performed, and the results of DLRG are listed here (**B**). The interplay of miRNA-126a-5p with CYP7A1 mRNA was examined, and the results of DLRG are listed here (**C**). A significant difference is indicated with the sign _*_ (*p* < 0.05)

In our experiment, a miRNA-291a-3p expression level was shown to be significantly decreased by RT–PCR, while miRNA-126a-5p was increased (Fig. 12A). miRNA-291a-3p and miRNA-126a-5p directly bound to and regulated AMPK and CYP7A1 mRNA, respectively. miRNA binding sites within the 3′-untranslated region (3′-UTR) of AMPK and CYP7A1 mRNA were also identified based on bioinformatics analysis. In line with this, AMPK expression was significantly upregulated by miRNA-291a-3p in 293 T cells, while CYP7A1 was significantly downregulated by miRNA-126a-5p. The dual luciferase reporter gene assay results showed that the relative luciferase activities were significantly decreased in comparison with the NK group after cotransfection into 293 T cells (Fig. 12B and C).

## Discussion

Based on the preparation and characterization of cigarette smoke components (CSCs), this experiment carried out CSC exposure studies to explore the effects of CSCs on rat lipid metabolism levels and their underlying metabolic mechanisms. It is known that more than a thousand chemicals have been identified and determined in cigarettes [19]. To accurately and precisely identify these chemicals, the neutral, acidic, and alkaline chemicals of CSC were prepared and analyzed by GC–MS according to published literature [20]. Among these chemicals, the content of nicotine in the alkaline component was the highest, accounting for 39.29% of this component. Some published articles have shown that smoking can result in obesity, lipid metabolism disorders, atherosclerosis and other metabolic diseases [21]. Then, some clinical studies have also suggested that nicotine was able to disturb the body’s metabolism process through multiple targets [22], which would result in metabolic diseases.

However, a great number of chemicals from cigarettes are decomposed at high temperature or volatilized during combustion, and these chemicals do not enter the body through smoking [23]. Then, the chemicals absorbed into blood circulation would likely dissolve in mucus secreted by mucosal epithelial cells. Therefore, the prepared chemicals (namely, CSC) in our experiment were absorbed into the circulation system to induce toxic effects. In this study, smoke exposure was able to slow the weight gain of rats and increase serum T-CHO, LDL-C, AST/ALT and AI levels. T-CHO, LDL-C, TG and HDL-C concentrations in serum reflect the blood lipid levels in rats, and AST/ALT levels reflect the degree of liver damage in rats [24]. The increase in AI levels may be an omen of the risk increase of atherosclerosis [ [25]]. These results revealed that CSC exposure was likely to cause dyslipidemia and fatty liver damage, which would eventually lead to atherosclerosis and fat liver disease in the long run.

The level of cholesterol in plasma is often used for the early diagnosis of clinical diseases such as hyperlipidemia, atherosclerosis, and type 2 diabetes [26, 27]. The level of ADP/ATP reflects the growth activity of cells, and it also partly reflects the energy metabolism status in tissue cells [28]. Trimethylamine oxide (TMAO) is a metabolite related to intestinal flora. In recent years, some scholars have confirmed that the increase in TMAO concentration in plasma often affects cholesterol homeostasis, lipid metabolism and the risk of atherosclerosis [ [29]]. Acetic acid, propanoic acid and butyric acid belong to the short-chain fatty acid family from intestinal flora, which is often used for maintaining intestinal function and health and regulating the metabolism of carbohydrates and lipids [30, 31]. In our study, the serum free cholesterol concentration, ADP/ATP ratio, and TMAO concentration in the CSC-exposed group were significantly increased (*P* < 0.05), and the contents of acetic acid, propanoic acid and butyric acid in the feces were significantly decreased (*P* < 0.05). These results indicated that CSC exposure causes an imbalance in cholesterol homeostasis, abnormal energy metabolism, lipid metabolism disorder, and gut microecological dysbiosis in rats.

Antioxidant capacity and clearance of free radicals are often used as important indexes to evaluate the body against oxidative and inflammatory damage, and these testing indicators mainly include catalase (CAT), superoxide dismutase (SOD) and some inflammatory factors (NF-κB and TNF-α) [32, 33]. In our study, the levels of serum NF-κB were significantly increased in CSC-exposed rats (*P* < 0.05), while the SOD level was decreased (*P* < 0.05). These results suggested that CSC exposure was able to weaken antioxidant capacity and increase inflammation in rats, which was in accordance with *Arnson Yoav* et al. and *Ma* et al.*’s* study [34, 35]. The CAT increase and MDA decrease, and these results can be attributed to the induction effect of H_2_O_2_ from mainstream cigarette smoke [36]. To further verify the toxicity of CSC exposure, the rat organ coefficients were calculated, and the test of liver morphology was performed by H&E staining and transmission electron microscopy (TEM). In this study, the liver and kidney organ indexes in the CSC exposure group were significantly increased (*P* < 0.05), which was mainly attributed to the rat body weight reduction. The fat tissue index was significantly decreased (*P* < 0.05), which was primarily caused by fat loss. These organ index results also suggested that CSC exposure was able to induce body weight and fat tissue loss, which may explain the phenomenon that women lost their weight by smoking [37, 38]. H&E staining showed that CSC exposure caused slight hepatocyte steatosis, and TEM observation showed that a partial subcellular structure of rat hepatocytes was injured by CSC. All these histological changes in the rat liver may be slightly attributed to mitochondrial damage to hepatocytes [39, 40]. Thus, an evaluation of mitochondrial function changes by CSCs should be developed. Mitochondrial membrane potential (MMP) is an important indicator for monitoring cell activity, mitochondrial membrane permeability and cell apoptosis [41]. In our study, there was a significant increase in the MMP of hepatocytes in the CSC exposure group (*P* < 0.05), indicating that CSC exposure had a significant effect on the mitochondrial membrane permeability of hepatocytes.

Moreover, there are nearly 100 trillion bacteria in the human intestine [42]. In recent years, studies have found that the gut flora has an important relationship with human metabolic diseases [43, 44]. At the phylum level, *Firmicutes* and *Bacteroidota* are the dominant bacterial communities in the human intestine [45]. Numerous studies have shown that *Firmicutes* in obese populations are more abundant, while the relative abundance of *Bacteroidota* is decreased [46]. In our experiment, at the phylum level, the abundance of *Firmicutes* was significantly increased in the CSC exposure group (*P* < 0.05), and the abundance of *Bacteroides* and *Spirochaetota* was significantly decreased (*P* < 0.05), which was in accordance with the literature. At the genus level, compared with the normal control group, the abundance of *Peptoclostridium*, *Turicibacter*, and *Clostridium* sensu stricto *1* was significantly increased in the CSC exposure group (*P* < 0.05), while the abundance of *Prevotella 9* and *Ruminococcaceae* was significantly decreased (*P* < 0.05), which suggested that changes in lipid metabolism induced by CSC exposure were closely associated with disturbance of the gut microbial community [47, 48]. However, in-depth mechanistic studies of the association between gut microbial composition and lipid metabolism are still lacking, although several mechanistic pathways have been clarified, including energy metabolism, immune system/gut barrier health, insulin resistance/satiety and bile acid metabolism [49, 50]. In our experiments, considering the changes in the ADP/ATP ratio and NF-κB concentration in CSC-exposed rats, it was likely that the changes in gut microbial composition induced by CSC exposure may cause energy metabolism abnormalities and immune system/gut barrier damage in CSC-exposed rats, which would subsequently result in lipid metabolism dysbiosis.

To further explore the mechanism of CSC exposure on blood lipid metabolism in rats, we used RT–PCR and Western blotting to detect the expression levels of lipid metabolism genes in the liver. 5′-AMP-activated protein kinase (AMPK) plays an important role in regulating glucose, fatty acid and protein metabolic pathways [51], and PRKAA2 is a gene encoding the α2 catalytic subunit of AMPK. Acetyl-CoA carboxylase β (ACC-2) plays a key role in fatty acid synthesis and oxidation pathways [52]. HMG-CoA reductase (encoded by the Hmgcr gene) is the rate-limiting enzyme for regulating cholesterol metabolism [53]. Cholesterol 7-hydroxylase (CYP7A1) is the rate-limiting enzyme of bile acid metabolism [54]. In this experiment, the phosphorylation levels of AMPK-ACC proteins in the CSC exposure group were significantly increased, while the expression levels of Hmgcr and CYP7A1 protein were significantly decreased, which suggested that CSC exposure was able to affect glucose and lipid metabolism by AMPK-ACC and CYP7A1 gene expression or phosphorylation. The activities of ACC and HMG-CoA reductase were both inhibited by their phosphorylation, so the acceleration of lipid catabolism and cholesterol synthesis were performed. Therefore, the increase in serum cholesterol may result from lipid catabolism and inhibition of bile acid production.

According to numerous reports [55, 56], a great deal of gene expression is markedly affected by miRNAs in the body. The expression of miRNA-291a-3p and miRNA-126a-5p was also significantly regulated by CSC exposure. Consequently, we speculated that miRNA-291a-3p and miRNA-126a-5p may be associated with the expression of certain corresponding target genes. This interplay was also predicted and validated by bioinformatics. In our study, the expression of AMPK and CYP7A1 was significantly suppressed by miRNA-291a-3p and miRNA-126a-5p by the use of the Dual Luciferase Reporter Gene Assay, which was partly consistent with some published reports [57, 58]. As far as this is concerned, lipid metabolism dysbiosis may be associated with miRNA level changes induced by CSC exposure. Further detailed mechanisms should be investigated in the future.

### Study strengths and limitations

The greatest strength of this study is to reveal the possible mechanism of lipid metabolism disorder and the related gene changes caused by CSCs exposure from the perspective of gut microbiota and serum exosomes in rats. However, the experiment only involved the animal level and extracellular vesicles, and the deeper mechanisms of interaction are uncertain among gut microbiota community, extracellular vesicles and body. Therefore, more evidences are needed to explore.

## Conclusions

CSC exposure was able to cause blood lipid metabolism dysbiosis in rats. The possible mechanism is that CSCs affect gut microecology- and lipid metabolism-related gene expression, which may be related to serum exosomes in rats. This study suggests that gut microbiota and serum exosomes are possible targets that affect lipid metabolism, and provides possible prevent and intervention for smoke-induced blood lipid metabolism disorders in clinic.

## Data Availability

The data in this paper are the real results of the experiment, and the original data can be provided by the authors.

## Abbreviations

CSC: Cigarette smoke components
TMAO: Trimethylamine oxide
AMPK: Adenylate-activated protein kinase
ACC: Acetyl-coenzyme A carboxylase
HMG-CoAR: 3-hydroxy-3-methyl glutaryl coenzyme A reductase
DLRG: Dual Luciferase Reporter Gene assay
RT–PCR: Reverse Transcription-Polymerase Chain Reaction
CYP7A1: Cholesterol 7α-hydroxylase
TNF-α: Tumor necrosis factor-alpha
ox-LDL: Oxidized low-density lipoprotein
NF-κB: Nuclear factor-kappa-B
HDL-C: High-density lipoprotein cholesterol
LDL-C: Low-density lipoprotein cholesterol
TC: Total cholesterol
TG: Triglycerides
AST: Aspartate aminotransferase
ALT: Alanine aminotransferase
CAT: Catalase
SOD: Superoxide dismutase
MDA: Malondialdehyde
FC: Free cholesterol
SCFA: Short-chain fatty acids
AI: Atherosclerosis index
HCL: Hydrochloric acid
TEM: Transmission electron microscope
SRA: Sequence Read Archive
